# The effects of Non3 mutations on chromatin organization in Drosophila melanogaster

**DOI:** 10.18699/vjgb-25-43

**Published:** 2025-06

**Authors:** A.A. Yushkova, A.A. Ogienko, E.N. Andreyeva, A.V. Pindyurin, A.E. Letiagina, E.S. Omelina

**Affiliations:** Institute of Molecular and Cellular Biology of the Siberian Branch of the Russian Academy of Sciences, Novosibirsk, Russia; Institute of Molecular and Cellular Biology of the Siberian Branch of the Russian Academy of Sciences, Novosibirsk, Russia; Institute of Molecular and Cellular Biology of the Siberian Branch of the Russian Academy of Sciences, Novosibirsk, Russia; Institute of Molecular and Cellular Biology of the Siberian Branch of the Russian Academy of Sciences, Novosibirsk, Russia; Institute of Molecular and Cellular Biology of the Siberian Branch of the Russian Academy of Sciences, Novosibirsk, Russia; Institute of Molecular and Cellular Biology of the Siberian Branch of the Russian Academy of Sciences, Novosibirsk, Russia

**Keywords:** nucleolus, NON3, HP1, CID, H3K9me2, chromatin, pericentromeric regions of chromosome, PEV, Su(var)205, Su(var)3-9, Drosophila, ядрышко, NON3, HP1, CID, H3K9me2, хроматин, прицентромерные районы хромосом, эффект положения, Su(var)205, Su(var)3-9, дрозофила

## Abstract

The nucleolus is a large membraneless subnuclear structure, the main function of which is ribosome biogenesis. However, there is growing evidence that the function of the nucleolus extends beyond this process. While the nucleolus is the most transcriptionally active site in the nucleus, it is also the compartment for the location and regulation of repressive genomic domains and, like the nuclear lamina, is the hub for the organization of inactive heterochromatin. Studies in human and Drosophila cells have shown that a decrease in some nucleolar proteins leads to changes in nucleolar morphology, heterochromatin organization and declustering of centromeres. This work is devoted to the study of the effects of Novel nucleolar protein 3 (Non3) gene mutations in D. melanogaster on the organization of chromatin in the nucleus. Previously, it was shown that partial deletion of the Non3 gene leads to embryonic lethality, and a decrease in NON3 causes an extension of ontogenesis and formation of a Minute-like phenotype in adult flies. In the present work, we have shown that mutations in the Non3 gene suppress the position effect variegation (PEV) and increase the frequency of meiotic recombination. We have analyzed the classical heterochromatin markers in Non3 mutants and shown that the amount of the HP1 protein as well as the modification of the histone H3K9me2 do not change significantly in larval brains and salivary glands compared to the control in Western blot analysis. Immunostaining with antibodies to HP1 and H3K9me2 did not reveal a significant reduction or change in the localization patterns of these proteins in the pericentromeric regions of salivary gland polytene chromosomes either. We analyzed the localization of the HP1 protein in Non3 mutants using DNA adenine methyltransferase identification (DamID) analysis and did not find substantial differences in protein distribution compared to the control. In hemocytes of Non3 mutants, we observed changes in the morphology of the nucleolus and in the size of the region detected by anti-centromere antibodies, but this was not accompanied by declustering of centromeres and their untethering from the nucleolar periphery. Thus, the NON3 protein is important for the formation/function of the nucleolus and is required for the correct chromatin packaging, but the exact mechanism of NON3 involvement in these processes requires further investigations.

## Abstract (ru)

Ядрышко – это крупная безмембранная субъядерная структура, где происходит биогенез рибосом. Однако известно все больше данных о том, что ядрышко выполняет и другие функции в клетке. Помимо того, что в ядрышке происходят активный синтез и процессинг рРНК, оно является компартментом, на периферии которого локализованы репрессированные участки генома. Таким образом, наряду с ядерной ламиной ядрышко выступает центром организации гетерохроматина. Исследования на клетках человека и дрозофилы показали, что снижение количества отдельных ядрышковых белков приводит к изменениям в морфологии ядрышка, организации гетерохроматина и декластеризации центромер. Данная работа посвящена изучению влияния мутаций в гене Novel nucleolar protein 3 (Non3) D. melanogaster на организацию хроматина в ядре. Ранее было показано, что делеция части гена Non3 приводит к эмбриональной летальности, а снижение количества белка NON3 – к замедлению онтогенеза и формированию Minute-подобного фенотипа у взрослых мух. В настоящей работе мы продемонстрировали, что мутации в гене Non3 супрессируют эффект положения мозаичного типа и увеличивают частоту мейотической рекомбинации. Общее количество классических маркеров гетерохроматина, белка HP1 и модификации гистона H3K9me2, в мозгах и слюнных железах личинок мутантов Non3 существенно не отличается от контроля, согласно оценке вестернблот анализом. Иммуноокрашивание антителами к HP1 и H3K9me2 также не выявило значительного изменения в количестве и паттерне локализации этих белков в прицентромерных районах политенных хромосом слюнных желез. Изучив локализацию белка HP1 у мутантов по гену Non3 с помощью метода DamID (DNA adenine methyltransferase identification), мы также не обнаружили значимых отличий в распределении белка по сравнению с контролем. В гемоцитах мутантов Non3 мы наблюдали изменение морфологии ядрышка и размера области, выявляемой антицентромерными антителами, но это не сопровождалось декластеризацией центромер и отхождением их от периферии ядрышка. Таким образом, белок NON3 важен для формирования/функционирования ядрышка и необходим для правильной упаковки хроматина, однако точный механизм участия NON3 в данных процессах требует дальнейшего изучения.

## Introduction

The nucleolus is a membraneless organelle that forms through phase separation in the nucleus. It is formed around nucleolus organizer regions (NORs), which contain ribosomal gene (rDNA) clusters encoding rRNAs (Pavlakis et al., 1979; Smirnov et al., 2016; Trinkle-Mulcahy, 2018). The main function of the nucleolus is the synthesis and processing of rRNA, production of the small 40S and large 60S ribosome subunits and ribosome assembly (Panse, Johnson, 2010). During interphase, the nucleolus can be divided into three compart- ments: the fibrillar center (FC), the dense fibrillar component (DFC), and the outer granular component (GC). The FC border is responsible for rDNA transcription, while the DFC and GC are involved in rRNA processing and the assembly of ribosomes, respectively. The FC is enriched in components of the RNA pol I machinery, such as the transcription factor UBF, whereas the DFC harbors various RNA-modifying enzymes and pre-rRNA processing factors including Fibrillarin (Boisvert et al., 2007; Boulon et al., 2010; Hernandez-Verdun et al., 2010; Razin, Ulianov, 2022). While the nucleolus is the most transcriptionally active site in the nucleus, it is also the compartment for the location and regulation of repressive genomic domains and, like the nuclear lamina, represents the hub for the organization of heterochromatin (Janssen et al., 2018; Quinodoz et al., 2018; Bersaglieri, Santoro, 2019; Iarovaia et al., 2019). Indeed, a shell of perinucleolar heterochromatin composed of silent rDNA, repetitive satellite DNA, heterochromatic regions from non-NOR-bearing chromosomes and pericentromeric/centromeric regions of chromosomes is often located close to the GC of the nucleolus (Németh, Längst, 2011).

Large segments of the eukaryotic genome are packaged in heterochromatin domains characterized by late replication and a low level of meiotic recombination. These domains containing arrays of repetitive sequences and transposable elements are enriched in H3K9me2/3 and harbor a small number of essential protein-coding genes. About one third of the Drosophila genome is considered heterochromatic, including the entire Y chromosome, most of the small chromosome 4 and the pericentric regions that cover 40 and 20 % of the X chromosome and the large autosomes, respectively (Grewal, Jia, 2007; Smith et al., 2007; Elgin, Reuter, 2013; Allshire, Madhani, 2018; Janssen et al., 2018). The best studied non-histone components of heterochromatin are HP1 encoded by the Su(var)205 gene (Lu et al., 2000) and Su(var)3-9 methyltransferase encoded by the gene of the same name (Schotta et al., 2002). HP1 is mainly found in the chromocenter, telomeres, chromosome 4, and in some sites on the chromosome arms (Meyer-Nava et al., 2020). Su(var)3-9 performs di- and trimethylation of H3K9, which is necessary for the specific binding of the HP1 protein (Rea et al., 2000; Schotta et al., 2002). Su(var)3-9 associates with the histone deacetylase HDAC1 (Czermin et al., 2001) and concerted histone deacetylation and methylation by a Su(var)3-9/ HDAC1-containing complex leads to permanent silencing of transcription in particular regions of the genome (Czermin et al., 2001). HP1 binding recruits additional Su(var)3-9 to methylate the adjacent nucleosome, which provides another binding site for HP1 in a self-propagating process (Schotta et al., 2003; Sentmanat, Elgin, 2012).

Centromeres are specialized domains of heterochromatin that provide the foundation for the kinetochore (Bloom, 2014). These multiprotein structures play an essential role during cell division by connecting chromosomes to spindle microtubules in mitosis and meiosis to mediate accurate chromosome segregation (Heun et al., 2006; Kyriacou, Heun, 2023). Centromeres are marked by the histone H3 variant centromere protein A (CENP-A, also called centromere identifier (CID) in Drosophila), which is necessary and sufficient for kinetochore activity (Bloom, 2014; Chang et al., 2019). Clustering and positioning of centromeres near the nucleolus is essential for the stable organization of pericentric heterochromatin in Drosophila (Padeken et al., 2013). Studies on human and Drosophila cells have provided evidence that a decrease in some nucleolar proteins can cause the repositioning of heterochromatin away from the nucleolar periphery and declustering of centromeres during interphase (Padeken et al., 2013; Rodrigues et al., 2023). The depletion of nucleolar proteins such as Nucleolin and Nucleophosmin led to changes in nucleolar morphology and heterochromatin organization (including decreased levels of H3K9me3 and HP1 foci at perinucleolar regions), as well as to mitotic defects (Olausson et al., 2014; Bizhanova, Kaufman, 2021). The decrease of Drosophila nucleolar protein Modulo (Nucleolin orthologue) led to declustering and untethering of centromeres from the nucleolar periphery (Padeken et al., 2013).

Previously, we demonstrated that Drosophila protein NON3 localizes to the nucleolus in larval brain cells. Null allele of the Non3 gene (Non3Δ600) causes early larval lethality, whereas viable combinations of hypomorphic alleles (Non3G4706/Non3259, Non3G4706/Non3197, Non3G4706/Non3310) result in Minute-like phenotype, which is manifested as prolonged development, poor viability and fertility, as well as abnormally short and thin bristles (Andreyeva et al., 2019). The NON3 protein belongs to the group of Brix domain-containing proteins, which are highly conserved from archaea to humans (Eisenhaber et al., 2001; Maekawa et al., 2018). The Brix domain is supposed to function as a structural hub for interactions with both proteins and RNA, mediated by its Nand C-terminal halves, respectively (Maekawa et al., 2018). The NON3 orthologous proteins are Ribosome production factor 2 (Rpf2) in S. cerevisiae (Morita et al., 2002), ARPF2 in A. thaliana (Maekawa et al., 2018) and RPF2 in humans (Hirano et al., 2009). All orthologues localize in the nucleolus. Human RPF2 and NON3 exhibit 66 % similarity and 47 % sequence identity (Gramates et al., 2017). Despite the fact that human RPF2 is an rRNA-interacting protein involved in pre-rRNA processing, it was isolated together with 172 proteins embedded in heterochromatic H3K9me3 domains in the course of proteomic analysis of purified H3K9me3-marked heterochromatin in human fibroblasts (Becker et al., 2017). The fact that RNA-binding proteins remain strongly enriched in H3K9me3-marked chromatin provides strong support for these proteins having a role in heterochromatin maintenance (Becker et al., 2017).

Here, we describe the role of the conserved NON3 protein in position-effect variegation (PEV) and show that Non3 mutations are weak suppressors of PEV. We also show that Non3Δ600 background slightly enhances meiotic recombination. However, neither immunostaining for HP1 nor genomewide DamID-seq mapping of HP1 binding to salivary gland polytene chromosomes reveals any substantial changes between the control and Non3 mutants. Finally, we provide evidence that Non3 mutations affect the size of the nucleolus and the region detected by anti-centromere antibodies in larval hemocytes, but do not affect the clustering of centromeres and their positioning relative to the nucleolus. Identification of new functions of nucleolar proteins may provide new insights into the functions of the nucleolus.

## Materials and methods

Fly stocks. All fly lines used in this work are presented in Table 1. Flies were raised and crossed on standard cornmeal agar media at 25 °C unless otherwise stated

**Table 1.**
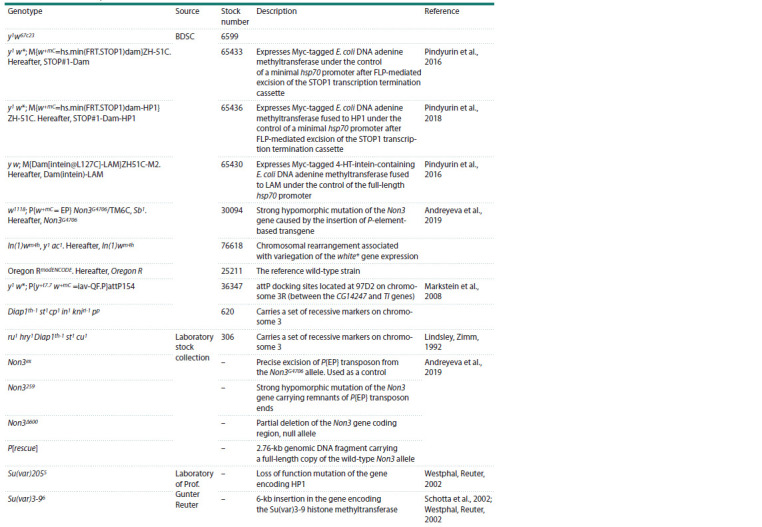
List of the used fly stocks Note. BDSC – Bloomington Drosophila Stock Center (Bloomington, IN, USA; flystocks.bio.indiana.edu). All Non3 mutations were balanced with the T(2;3)TSTL, CyO: TM6B, Tb1.

Generation of the sgs3-FLP transgenic construct and Drosophila germline transformation. To make the sgs3-FLP construct, a cassette consisting of a 1345-bp genomic DNA fragment [chr3L:11510890–11512234, but with 11510939A>G, 11511517G>T, 11511567A>C and 11511624T>A substitutions; the coordinates are from Release 6 of the D. melanogaster genome assembly (Hoskins et al., 2015)] spanning the salivary gland-specific Sgs3 gene promoter (Biyasheva et al., 2001; McPherson et al., 2024; Suárez Freire et al., 2024) and the FLP recombinase coding sequence were cloned upstream of the SV40 poly(A) sequence of the pattB vector (DGRC Stock 1420; https://dgrc.bio.indiana.edu//stock/1420; RRID:DGRC_1420). Details of plasmid construction are available upon request. The sgs3-FLP construct was integrated into the genome at the attP154 site (chromosome 3R) (Petersen, Stowers, 2011) mainly as described previously (Bischof et al., 2007) using the fly stock BDSC #36347.

Eye pigment analysis. Red eye pigment extraction and analysis were performed as described previously (Сonnolly et al., 1969) with the following modifications. Adult flies were aged for 3 days at 18 °C before measurement. For analysis, we took thirty heads of each sex per genotype. The optical density was measured at 480 nm using a Multimode Microplate Reader (Tecan SPARK® 10M).

Meiotic recombination analysis. We counted crossingover frequencies along chromosome 3 using two different fly strains (##306, 620) carrying recessive marker mutations and the Non3Δ600 mutation. Non3ex was used as control. For each studied chromosomal region, the frequency of meiotic recombination between markers was calculated by dividing the number of recombinant progeny by the total number of flies analyzed.

Western blotting. Immunoblotting was performed as described earlier (Andreyeva et al., 2019). The following primary antibodies were used: mouse anti-β-Tubulin (1:800; BX69 (Tavares et al., 1996), kindly provided by Prof. Harald Saumweber), mouse anti-Non3 (1:5,000 (Andreyeva et al., 2019)), mouse anti-HP1 (1:800, Developmental Studies Hybridoma Bank (DSHB) C1A9), mouse anti-H3K9me2 antibody (1:400, Abcam 1220). The primary antibodies were detected with HRP-conjugated goat anti-mouse IgG (1:3,500; Life Technology G-21040) and images were captured using an Amersham Imager 600 System (GE Healthcare). Band intensities were analyzed using ImageJ. The intensity of each band was normalized with the intensity of the corresponding loading control.

Immunostaining and microscopy. Indirect immunofluorescence (IF) staining of polytene chromosomes, whole-mount salivary glands and hemocytes was carried out as described previously (Andreyeva et al., 2017; Tracy, Krämer, 2017; Meyer-Nava et al., 2021). The following primary antibodies were used: rabbit anti-HP1 (1:100, kindly provided by Prof. Peter Verrijzer), mouse anti-NON3 (1:50 (Andreyeva et al., 2019)), mouse anti-H3K9me2 (1:100, Abcam 1220), mouse anti-Fibrillarin antibody (38F3; 1:100, Thermo MA1-22000), rabbit anti-CID (1:200, Abcam 10887). The primary antibodies were detected with goat anti-rabbit IgG (H+L) highly cross-adsorbed secondary antibody, Alexa Fluor™ 568 (1:500, Thermo Scientific A-11036), and goat anti-mouse IgG (H+L) cross-adsorbed secondary antibody, Alexa Fluor™ 488 (1:500, Thermo Scientific A-11001). Samples were imaged using a Zeiss Axio Imager M2 (Carl Zeiss) and a confocal microscope LSM 710 (Carl Zeiss). Optical sections were combined using the LSM Image Browser version 4.2 software (Carl Zeiss).

Image analysis. To measure relative fluorescence intensity of the HP1 protein and H3K9me2 histone modification on polytene chromosomes and whole-mount salivary glands, the fluorescent signals recorded separately as grayscale digital images were pseudocolor-coded and merged using the ImageJ program. In the case of whole-mount salivary glands, we analyzed only corpuscular cells. Hemocyte image analysis was done using Zeiss LSM Image Browser 4.2.0.121 software. Centromere clusters were defined by anti-CID antibodies with an individual center of gravity. Briefly, a sum projection over 4 optical z-sections (0.35 μm each) was created for each centromere foci and nucleolus centered around the brightest pixel of the structure. Distances of centromeres to the nearest nucleolus were measured by drawing a line from the center of the centromere to the edge of the nucleolus. Areas of the nucleolus, centromeres and nuclei were measured by outlining their boundaries and calculating the areas of the resulting polygons. 35 hemocytes of Oregon R, 39 hemocytes of Non3Δ600/Non3259 and 39 hemocytes of Non3Δ600/Non3G4706 third-instar larvae were analyzed.

DamID-seq procedure. Fly genotypes used for DamID experiments are listed below in the relevant section. Each experiment was performed in two technical replicates with 60 salivary glands in each replicate dissected from third-instar larvae. Isolation of genomic DNA from the collected material and the entire DamID procedure were performed as previously described (Pindyurin, 2017). To remove DamID adapters from the PCR-amplified Dam-methylated DNA fragments, the latter were digested with DpnII restriction enzyme. After that, the size of the DNA fragments was reduced to a range of 150–450 bp by ultrasonic fragmentation using the Bioruptor Pico Sonication system (Diagenode). Libraries for NGS were prepared using the TruSeq protocol (Illumina).

Illumina NGS and data analysis. Sequencing of the samples was carried out on the Illumina MiSeq 2 × 75 bp platform using the MiSeq Reagent Kit v3 150 cycles (Illumina). The obtained fastq files contained ~1–2 million reads for each sample. The quality analysis of the raw data was performed using the FastQC tool (https://www.bioinformatics.babraham. ac.uk/projects/fastqc/). Subsequent bioinformatic analysis of DamID-seq data was done as described earlier with minor modifications (Pindyurin et al., 2018). Briefly, sequencing reads from two technical replicates of Dam or Dam- HP1 samples were adapter clipped and uniquely mapped to the dm6 genomic assembly by “bowtie2” (Langmead et al., 2009). Reads were counted by “HTSeqcount” software (Anders et al., 2015) in GATC genomic fragments. Next, read counts were merged between the replicates, as they were highly correlated (the Pearson correlation coefficient = 0.93–0.97). The resulting read counts of Dam or Dam-HP1 samples were converted to reads per million (RPM), and then Dam-HP1 values were normalized to those of the Dam and log2 transformed. Finally, quantile normalization was applied.

## Results

**Fig. 1.**
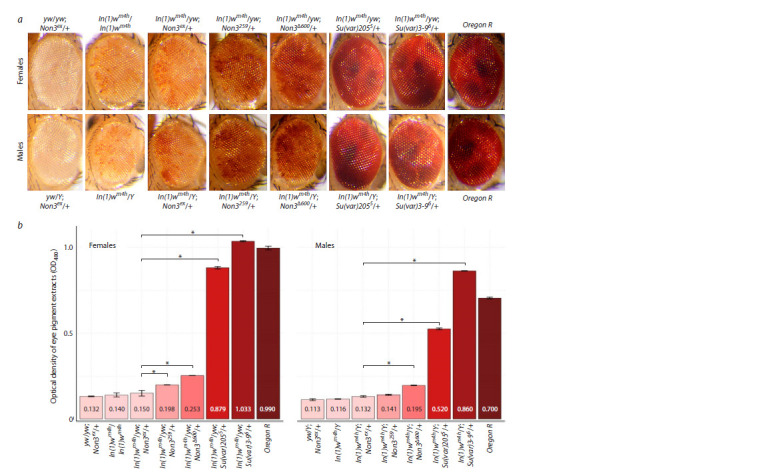
Suppression of PEV by the Non3 mutations at 18 °C. a – Eye pigmentation of control and mutant flies. The most characteristic images for each of the indicated genotypes are provided. b – Quantification of PEV phenotype of adult flies based on the concentration of red eye pigment. Data are graphically represented as a histogram for three measurement points for each genotype. The y-axis reflects the optical density (OD480) of the eye pigment extracts from the flies of the indicated genotypes. Numbers inside the columns indicate the average pigment optical density for each genotype. * Significance level p < 0.05, pairwise t-test.

We investigated the role of NON3 in nucleolar morphology, heterochromatin organization, and centromere localization. First, we examined whether Non3 mutants could modify PEV. In Drosophila, PEV assay has been extensively employed to study heterochromatin formation (Elgin, Reuter, 2013). We used In(1)wm4 inversion, in which the normally euchromatic white+ gene responsible for eye pigmentation is placed close to the pericentric heterochromatin due to chromosomal inversion and becomes silent in some cells (Cooper, 1959). To test whether Non3 mutations modify PEV, we genetically combined inversion In(1)wm4h with the following Non3 alleles: Non3ex (control), Non3259 (strong hypomorphic mutation), and Non3Δ600 (null allele of the Non3 gene) (Andreyeva et al., 2019). The Su(var)2055 and Su(var)3-96 mutations, known PEV suppressors (Eissenberg et al., 1992; Schotta et al., 2002), were used as references. The yw/yw; Non3ex/+ and Oregon R flies were used as negative and positive controls for absence/ presence of eye pigment, respectively (Fig. 1a). PEV can be modified by a variety of factors. The temperature of development and the amount of heterochromatin within the genome were the first factors shown to affect the extent of variegation (Elgin, Reuter, 2013). We carried out experiments separately for males and females at 18 °C. The visual inspection of fly eyes showed that both the Non3259 and the Non3Δ600 mutations combined with In(1)wm4h lead to an increase in white+ expression compared to control (Fig. 1a).

To quantitatively measure the effects, we performed extraction and measurement of the eye pigment. For example, the Non3259 mutation (In(1)wm4h/yw; Non3259/+) resulted in a 1.32- (for females) and 1.06- (for males) fold increase in the eye pigmentation level compared to control (In(1) wm4h/ yw; Non3ex/+). The Non3Δ600 mutation (In(1)wm4h/yw; Non3Δ600/+) resulted in a 1.67- (for females) and 1.47- (for males) fold increase in the eye pigmentation level compared to control (In(1)wm4h/yw; Non3ex/+) (Fig. 1b). Taken together, these results demonstrate that Non3 mutations are suppressors of PEV, although their influence is significantly lower (~4 times for both females and males) than that of the Su(var)2055 and Su(var)3-96 mutations. Generally, the results confirmed the visual observations of fly eyes (Fig. 1a).

**Fig. 2.**
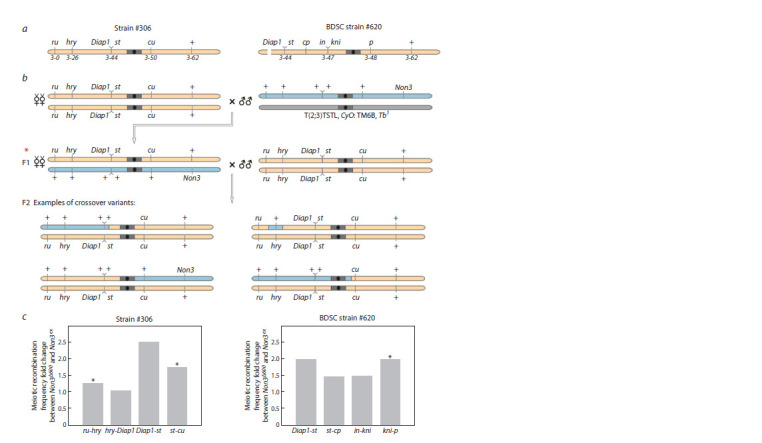
Mutations in the Non3 gene increase the meiotic recombination frequency. a – Sсhematic presentation of strains ##306 and 620 carrying a set of recessive mutations in chromosome 3 and used for analysis of meiotic recombination frequencies. The numbers indicate the localization of recessive mutations on the genetic map according to (Gramates et al., 2017). Pericentromeric heterochromatin and the centromere are shown by a gray rectangle and a black circle, respectively. b – Experimental setup. The #306 strain is shown as an example. The initial cross involves the homozygous strain #306 and the strain carrying any of the Non3 alleles (Non3ex or Non3Δ600). Virgin F1 females (red asterisk), in the gonads of which crossing-over takes place, are crossed with males of the strain #306. Several examples of F2 crossovers are shown. c – Effect of the heterozygous Non3Δ600 mutation on the frequency of meiotic recombination at the studied chromosomal regions as compared to the Non3ex control. * Significance level p < 0.01 by Fisher’s exact test.

Next, we decided to perform meiotic recombination analysis within the euchromatin and pericentromeric regions in Non3 mutants. Normally, recombination in pericentromeric heterochromatin is almost absent and strongly suppressed in adjacent euchromatic regions (Baker, 1958; Westphal, Reuter, 2002). However, the dominant effects of suppressors of PEV on crossing-over in the pericentromeric regions were shown for some mutations. For example, for double mutants Su(var)2055 and Su(var)3-96, the meiotic recombination frequency in the pericentromeric regions between the marker genes kni and p was increased (Westphal, Reuter, 2002). We used two different strains (##306, 620) carrying viable recessive genetic markers on chromosome 3 (Fig. 2a) and measured the crossing-over frequencies between them in a Non3 mutant background (Fig. 2b, Supplementary Tables S1, S2)1. In Figure 2, we presented crossings for strain #306 but not for strain #620, since it was similar to #306. We analyzed 2,380 crossover flies for strain #306 and 3,079 flies for strain #620 (Table 2, Supplementary Tables S1, S2). For strain #306, we showed that the presence of one copy of the Non3Δ600 allele in the genome leads to a statistically significant 1.24- and 1.71-fold increase in recombination frequency in the euchromatin region between the marker genes ru and hry and in the pericentromeric regions between the marker genes st and cu, respectively. For strain #620, we observed a statistically significant 1.95-fold increase in recombination frequency in the pericentromeric region between the marker genes kni and p (Fig. 2c, Table 2). The results suggest a possible role of NON3 in maintenance of integrity and stability of the euchromatin and pericentromeric regions.


Supplementary Materials are available in the online version of the paper: https://vavilovj-icg.ru/download/pict-2025-29/appx14.pdf


**Table 2.**
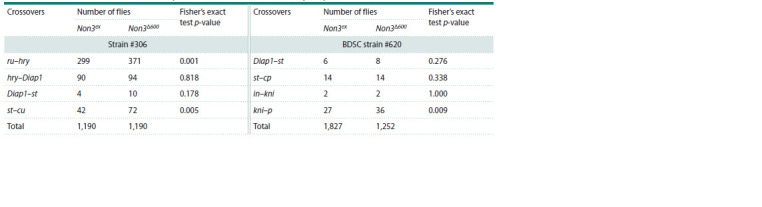
The number of F2 crossover flies analyzed for meiotic recombination frequency

**Fig. 3.**
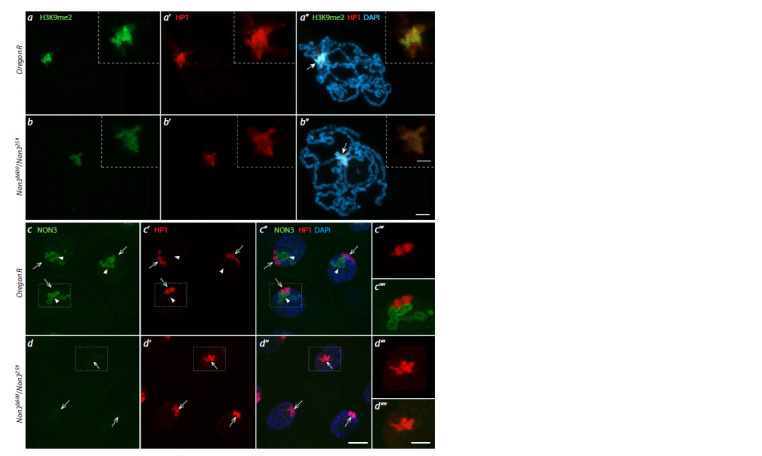
The levels of the HP1 protein and H3K9me2 histone modification in larval salivary glands of Non3 mutants. a and b – Immunofluorescence images of polytene chromosome staining from wild-type Oregon R (a–a’’) and Non3Δ600/Non3259 mutant (b–b’’) larvae co-stained with anti-HP1 and anti-H3K9me2 antibodies. The intensities of HP1 and H3K9me2 signals are slightly reduced in mutants compared to control. Arrows with a filled arrowhead indicate the chromocenter. Images in dotted frames represent magnification of the chromocenter. c and d – Confocal microscopy (maximum projection) of salivary gland nuclei from wild-type Oregon R (c–c’’) and Non3Δ600/Non3259 mutant (d–d ’’) larvae co-stained with anti-HP1 and anti-NON3 antibodies. There is no detectable NON3 signal in Non3Δ600/Non3259 mutants compared to control, but some increase in the fluorescence level of the HP1 protein in Non3 mutants vs control is detected. Arrows indicate the HP1 signal, arrowheads show nucleoli visualized by anti-NON3 antibodies. Fragments marked by a dotted rectangle are shown with larger magnification in (c’’’, c’’’’, d ’’’, d ’’’’). DNA is visualized by DAPI (blue), nucleolus by anti-NON3 antibodies (green), H3K9me2 histone modification, by the corresponding antibodies (green), the HP1 protein by the corresponding antibodies (red). Scale bar for all images except magnification is 20 μm, for enlarged fragments of microphotographs, 10 μm.

**Fig. 4.**
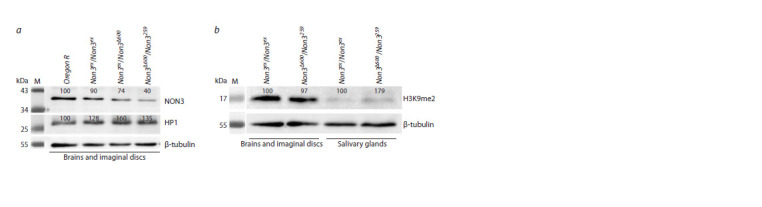
The levels of the HP1 and NON3 proteins, as well as H3K9me2 histone modification in larval tissues from Non3 mutants a – Western blot from larval brains with adjacent imaginal discs showing that in Non3Δ600/Non3259 mutants, the level of the NON3 protein is substantially reduced compared to the Oregon R and Non3ex/Non3ex controls. b – Western blot from larval brains with adjacent imaginal discs and salivary glands. H3K9me2 histone modification is not reduced in both tissue types in Non3Δ600/Non3259 mutants compared to the Non3ex/Non3ex controls. The numbers show the intensity of each band normalized to the intensities of the corresponding loading control taken as 100 %. M – Prestained Protein Ladder. The protein level of β-tubulin is shown as a loading control.

We sought to understand whether NON3 is required for chromosomal localization of the heterochromatin components. For that, the Non3 mutant and wild-type Oregon R squash preparations of polythene chromosomes were immunostained with anti-HP1 and anti-H3K9me2 antibodies. No difference in the protein binding patterns at the chromocenter between the mutant and the control background was found, but the intensity of the signals was reduced by 27.6 and 23.0 % for HP1 and H3K9me2, respectively, in Non3 mutants (N = 41) compared to control (N = 81) (Fig. 3a, b). However, when whole-mount salivary glands were immunostained with anti-HP1 antibodies, we even found an increase in the HP1 intensity of 36.0 % in Non3 mutants (N = 82) in comparison with control (N = 75) (Fig. 3c, d). The data of whole-mount salivary glands’ immunostaining was in accordance with Western blotting analysis of total protein levels from larval salivary glands and brains with adjacent imaginal discs, which showed that the total levels of the HP1 protein (Fig. 4a) and H3K9me2 histone modification (Fig. 4b) were slightly increased in Non3Δ600/Non3259 mutants compared to control.

**Fig. 5.**
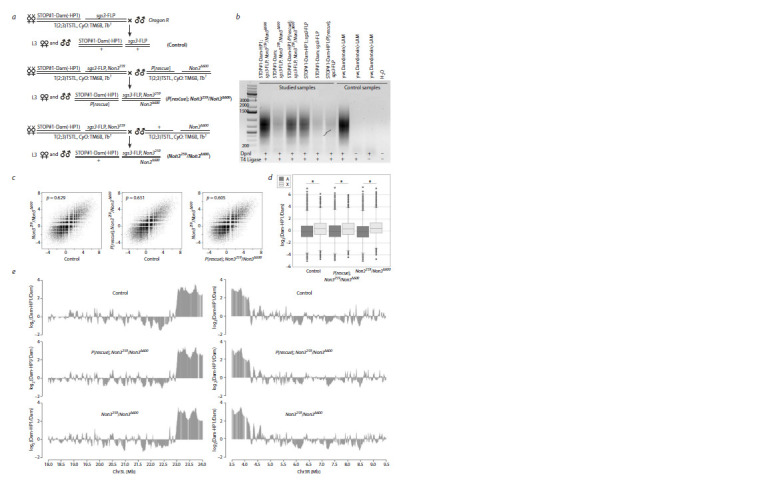
FLP-inducible Drosophila DamID system in salivary glands of the Non3 mutant and control third-instar larvae. a – Genetic crosses used to activate STOP#1-Dam(-HP1)-containing transgenes. b – Methylation detected in genomic DNA isolated from larval salivary glands. Specificity of amplification of the methylated GATC fragments was confirmed by the ‘-DpnI’ and ‘-T4 DNA ligase’ control reactions. Dam(intein)-LAM flies were used as a positive control; the banded pattern is derived from mitochondrial DNA. c – Genome-wide correlation between the studied datasets (p – Pearson’s correlation coefficient). d – Box plots showing distributions of log2(Dam-HP1/Dam) values in the non-repetitive parts of the X chromosome (light gray) and autosomes (dark gray) in the studied samples. Wilcoxon rank sum test was used for pairwise comparison of distributions on the X chromosome vs autosomes, * p-value < 2.2 · 10–16. e – Mutation in the Non3 gene does not substantially affect the HP1 binding profile. Representative 6.0-Mb fragments of chromosomal arms 3L and 3R are shown. A running mean algorithm (a sliding window of 50 GATC fragments, one fragment per step) was applied to the HP1 binding data.

Next, we investigated the distribution of the HP1 protein in the salivary gland polytene chromosomes of Non3 mutants using the DamID approach. To generate DamID profiles of HP1, we used the FLP-inducible STOP#1-Dam system (Pindyurin et al., 2016). Expression of Dam only or Dam-HP1 construct in larval salivary glands was activated by FLP recombinase expressed under the control of the sgs3 promoter, which is specifically active in this tissue (Biyasheva et al., 2001; McPherson et al., 2024; Suárez Freire et al., 2024). To achieve that, the sgs3-FLP transgene was integrated at the 97D2 region and its activity was indeed detected in larval salivary glands but not in whole adult flies (Supplementary Fig. S1). Next, we combined the sgs3-FLP transgene with the Non3259 mutation on the same chromosome. Then, we generated larvae of the following genotypes: STOP#1-Dam (-HP1)/+; sgs3-FLP, Non3259/Non3Δ600 or STOP#1-Dam (-HP1)/+; sgs3-FLP/+ or STOP#1-Dam (-HP1)/P[rescue]; sgs3-FLP, Non3259/Non3Δ600 (Fig. 5a). Subsequent amplification of Dam-methylated fragments of the salivary gland genome was performed as previously described (Pindyurin et al., 2017). The high specificity of the amplification procedure was confirmed by gel electrophoresis showing substantially more mePCR products in experimental samples compared to negative controls (Fig. 5b). DamID-derived libraries were subjected to Illumina sequencing and analyzed as described previously (Pindyurin et al., 2018). The correlation between control, Non3 mutants carrying one copy of the rescue construct (P[rescue]) and Non3 mutant-only samples across the entire genome showed no significant difference (Fig. 5c). All three samples demonstrated the increased binding of HP1 protein to chromosome X in comparison with autosomes (Fig. 5d), which is in good agreement with previous reports for larval brains, neurons, glia, fat body and Kc167 cells (Pindyurin et al., 2018). Comparison of DamID profiles obtained for all three samples did not reveal substantial changes either at the pericentromeric regions or at any other parts of the chromosomes (Fig. 5e, Supplementary Figs. S2, S3), suggesting that NON3 is not essential for the binding of HP1 to chromatin.

**Fig. 6.**
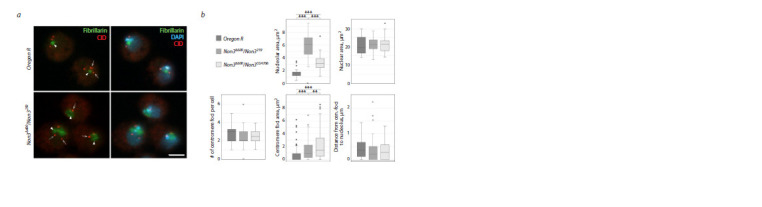
The lack of the NON3 protein in Non3 mutants leads to an increase in the size of the nucleolus and centromeres a – Immunofluorescence images of fixed Drosophila hemocytes during interphase, isolated from Oregon R (control) and Non3Δ600/Non3259 mutants, show the relative position of the centromere (CID) and nucleolus (Fibrillarin) in cells. Arrows indicate the centromeres, arrowheads, the nucleolus. b – Quantification of the nucleolar area, number of centromere foci per cell, distance from centromere foci to the nucleolus, the centromere foci area and nuclear area in hemocytes of Oregon R, Non3Δ600/Non3259 and Non3Δ600/ Non3CG4706 third-instar larvae. ** Significance level p < 0.01, *** p < 0.001, Student’s t-test. Nuclear DNA is visualized by DAPI (blue), nucleolus by anti-Fibrillarin antibodies (green), and centromere by anti-CID (red). Scale bar for all images: 5 μm.

To understand if NON3 has a role in the tethering and clustering of centromeres, we isolated hemocytes from Drosophila third-instar larvae and analyzed the number of centromeres per cell and their localization relatively to the nucleolus. Drosophila diploid larval hemocytes possess 8 centromeres revealed as 2–3 individual centromere foci (Padeken et al., 2013), which have been described to cluster together and associate with the periphery of the nucleolus (Fig. 6a) (Padeken, Heun, 2013; Padeken et al., 2013). We observed that in Non3Δ600/Non3259 and Non3Δ600/Non3G4706 mutant interphase hemocytes, the size of the nucleolus was increased (Fig. 6a, b), while the size of the nucleus was not changed. In wild-type hemocytes, the mean size of the nucleolus was 1.56 ± 0.10 μm2, in Non3Δ600/Non3259, 5.82 ± 0.32 μm2, and in Non3Δ600/Non3G4706, 3.22 ± 0.19 μm2. In mutant hemocytes, we did not detect any increase in individual centromere foci per cell or any significant dissociation of centromeres from the nucleolar periphery, but observed an increase in the size of the regions detected by anti-centromere antibodies (Fig. 6b). In wild-type hemocytes, the mean size of the analyzed regions was 0.07 ± 0.01 μm2, while in Non3Δ600/Non3259 mutants, it was 0.140 ± 0.02 μm2, and in Non3Δ600/Non3G4706 mutants, 0.216 ± 0.02 μm2.

## Discussion

Increasing evidence suggests that the function of the nucleolus goes beyond ribosome biogenesis. Among many other functions, the nucleolus is considered as the hub for the organization of inactive chromatin in the cell (Quinodoz et al., 2018). In this study, we aimed to understand the role of Drosophila Non3 mutations in chromatin organization. We have found that Non3 is a weak suppressor of PEV (In(1)wm4h) implicating NON3 directly or indirectly in the maintenance of normal chromatin structure during eye development. The involvement of nucleolar proteins in chromatin compaction is not surprising. Previously, it was shown that mutants of modulo display a suppressor effect on PEV. The Modulo protein binds DNA directly and may serve to anchor multimeric complexes, promoting chromatin compaction and silencing (Garzino et al., 1992). NON3 does not have a predicted DNA-binding domain (https://www.uniprot.org/uniprotkb/Q9VEB3/entry); therefore, we suggest that it might be associated with some unknown factors to form condensed chromatin.

In addition to the effect on PEV, we have shown that the frequency of meiotic recombination is increased when one copy of Non3 is missing from the genome. In 2002, T. Westphal and G. Reuter conducted a large-scale screen to assess the effects of PEV suppressor genes on crossing-over frequency in the pericentromeric regions of chromosomes. It was shown that 16 mutations in the Su(var) genes have a significant effect on increasing the frequency of meiotic recombination in the region of chromosome 3 between the kni–p markers. Heterozygous combination of the Su(var)2055/+; Su(var)3-96/+ mutations increased recombination at this chromosomal region by 1.4 times compared to control (Westphal, Reuter, 2002). These results confirmed that the frequency of crossover events can be controlled at the level of chromatin structure. We showed that the deletion of one functional copy of Non3 in the genome increased the frequency of meiotic recombination in the kni– p and st–cu regions compared to the control Non3ex allele. Moreover, an increased frequency of meiotic recombination was also observed in the euchromatic ru–hry region of chromosome 3 located far away from the centromere (Table 2). Altogether, our results indicate that a single functioning copy of Non3 is not enough for maintenance of normal chromatin structure; it is especially evident in the pericentromeric regions of chromosome 3.

Since Non3 mutants suppress PEV and enhance meiotic recombination in the pericentromeric regions of chromosome 3, we wondered whether the localization pattern of the main heterochromatin components, HP1 and H3K9me2, is somehow affected in Non3 mutants. No substantial changes were found in their localization patterns on salivary gland polytene chromosomes, but we noticed a slight decrease in HP1 and H3K9me2 signals in a Non3 mutant background using acetic acid fixation (Fig. 3a, b). However, immunostaining with formaldehyde fixation and/or immunoblotting of whole salivary glands did not detect a decrease in amounts of these proteins within the cells (Fig. 3c, d, Fig. 4). The difference in HP1 signal intensities seen using various fixation methods can be explained by the fact that acetic acid is prone to extract histones from the tissues (Dick, Johns, 1968; Johansen et al., 2009). Since NON3 seems to play some role in maintenance of integrity and stability of the pericentromeric regions, acetic acid fixation may extract histones more extensively in Non3 mutant tissues. This may be the reason for the observed differences.

We also examined HP1 binding in the polytene chromosomes of Non3 mutants with higher resolution using the DamID approach, but did not detect substantial differences compared to control animals either in the pericentromeric regions of chromosome 3 or somewhere else in the genome (Fig. 5e, Supplementary Figs. S2, S3). Thus, the effects on PEV and meiotic recombination in pericentromeric chromosomal regions observed in Non3 mutants do not seem to be due to changes in HP1 localization pattern.

Since there is a connection between clustering and positioning of centromeres near the nucleolus and stable organization of pericentric heterochromatin (Padeken et al., 2013), we analyzed the number and localization of centromeres using Drosophila third-instar larval hemocytes. We found that in Non3 mutants, the number of centromeres (CID foci) in interphase cells is not different from control. We also did not detect any untethering centromeres from the periphery of the nucleolus in Non3 mutants. However, we observed an increase in the size of the nucleolus and centromeres in Non3Δ600/ Non3259 and Non3Δ600/Non3G4706 mutant hemocytes (Fig. 6). Previously, it was shown that reduction of the NON3 orthologue protein ARPF2 leads to the redistribution of Fibrillarin and Nucleolin from the nucleolus to the nucleoplasm (Choi et al., 2020). In another study, FCs and DFCs were delocalized to the periphery of the GC upon a significant decrease in the levels of Nucleolin protein in HeLa cells (Ugrinova et al., 2007). In the case of Non3 mutants, we cannot exclude that there is no enlargement of the nucleolus but Fibrillarin has moved beyond the DFC compartment. It is interesting to note that the disappearance of Fibrillarin was earlier observed in Non3 mutant larval brain cells (Andreyeva et al., 2019). Such a discrepancy with the findings of the present study might be caused either by cell type-specific peculiarities or differences in the fixation methods used. We also observed an increase in centromere size in Non3 mutants (Fig. 6b). Centromeres are generally flanked by heterochromatin (Kapoor et al., 2015) and it was shown earlier that flanking heterochromatin is a prerequisite for maintaining centromeres (Henikoff et al., 2001). Therefore, we suggest that the increase in centromere size in Non3 mutants may be associated with the role of NON3 in maintenance of integrity and stability of the pericentromeric regions of chromosomes.

## Conclusion

Thus, we analyzed the effects of Non3 mutants on chromatin organization in the nuclei of various Drosophila tissues. We have shown that Non3 mutants suppress PEV, enhance meiotic recombination in the euchromatin and pericentromeric regions of chromosome 3, however, this does not accompanied by any significant changes in the amount or distribution of classical heterochromatin markers: the HP1 protein as well as the modification of the histone H3K9me2. In Non3 mutants, we observed an increased size of both, the nucleoli and the region detected by anti-centromere antibodies. However, we did not detect centromere declustering or their detachment from the nucleolar periphery. Thus, we suggest that the NON3 protein is important for the formation/function of the nucleolus and is required for the correct chromatin packaging, but the exact mechanism of NON3 involvement in these processes requires further study.

## Conflict of interest

The authors declare no conflict of interest.
